# Impact of Stress Hyperglycemia on Long‐Term Outcomes in Patients With Acute Kidney Injury Requiring Continuous Renal Replacement Therapy: A Nationwide Cohort Study

**DOI:** 10.1155/jdr/7769851

**Published:** 2026-03-26

**Authors:** Junseok Jeon, Danbee Kang, Hyejeong Park, Minhyung Kim, Kyungho Lee, Jung Eun Lee, Wooseong Huh, Juhee Cho, Hye Ryoun Jang

**Affiliations:** ^1^ Division of Nephrology, Department of Medicine, Samsung Medical Center, Sungkyunkwan University School of Medicine, Seoul, Republic of Korea, skku.edu; ^2^ Center for Clinical Epidemiology, Samsung Medical Center, Sungkyunkwan University School of Medicine, Seoul, Republic of Korea, skku.edu

**Keywords:** cardiovascular disease, continuous renal replacement therapy, critical illness, hyperglycemia, new-onset diabetes

## Abstract

**Aim:**

Stress hyperglycemia, occurring during stressful conditions in patients without preexisting diabetes, is associated with poor outcomes in critically ill patients. We investigated its impact on long‐term outcomes in patients with acute kidney injury (AKI) requiring continuous renal replacement therapy (CRRT).

**Materials and Methods:**

This retrospective cohort study included 11,013 adult patients without preexisting diabetes who received CRRT for ≥3 days between 2010 and 2019 and were discharged alive using the National Health Insurance Service database of South Korea. Stress hyperglycemia was defined as receiving oral hypoglycemic agents (OHAs) or insulin for at least 7 days.

**Results:**

Among survivors to discharge, 2409 (21.9%) patients received OHA/insulin treatment during hospitalization. Stress hyperglycemia was associated with a lower risk of mortality (adjusted hazard ratios [HR] = 0.92, 95% confidence interval [CI] = 0.86–0.99). However, the hyperglycemia group had a higher risk of acute myocardial infarction (MI) (HR = 1.53, 95% CI = 1.11–2.12) and revascularization procedures (adjusted HR = 1.41, 95% CI = 1.02–1.94). Furthermore, among patients who survived for at least 1 year and did not receive diabetes treatment within 1 year (*n* = 5100), the hyperglycemia group had a significantly higher risk of developing new‐onset diabetes after discharge (HR = 10.10, 95% CI = 6.92–14.73).

**Conclusions:**

In critically ill patients with AKI requiring CRRT and without preexisting diabetes, stress hyperglycemia was associated with a decreased risk of mortality but an increased risk of coronary events and new‐onset diabetes after discharge.

## 1. Introduction

Stress hyperglycemia, during acute illness in patients without preexisting diabetes, affects up to 38% of hospitalized patients [1, 2] and is commonly treated with glucose‐lowering medications in clinical practice. Several factors contribute to hyperglycemia in acute illness, including stress hormones. Stress hyperglycemia is associated with poor outcomes in hospitalized patients across various clinical settings [2–5]. Notably, hyperglycemia in patients without diabetes appears to be linked to even higher in‐hospital mortality rates compared to diabetic patients [2, 4].

Patients with acute kidney injury (AKI) face a unique risk for both hypoglycemia and hyperglycemia. Kidneys play a role in both glucose and insulin metabolism [6]. Impaired kidney function, as kidneys metabolize insulin, increases the risk of hypoglycemia by extending insulin half‐life [7, 8]. In one study of critically ill patients, continuous renal replacement therapy (CRRT) was an independent risk factor for hypoglycemia [9]. Conversely, insulin resistance, common among patients with AKI, increases the risk of hyperglycemia and mortality [10]. Therefore, factors influencing hyperglycemia and its outcomes in patients with AKI might differ from those in patients without AKI.

Beyond short‐term effects, hyperglycemia during acute illness is associated with an increased risk of long‐term mortality or new‐onset diabetes in survivors [11–14]. However, the impact of hyperglycemia on long‐term patient outcomes, particularly in severe AKI requiring CRRT, remains unclear. This study aimed to investigate the overall impact of stress hyperglycemia on patient outcomes in critically ill patients undergoing CRRT for AKI with a nationwide cohort.

## 2. Materials and Methods

### 2.1. Data Source

This retrospective cohort study obtained data from the Korean National Health Insurance Service (NHIS) database, covering ~97% of the South Korean population. The remaining 3% are covered by the Medical Aid Program [15]. The NHIS database comprises modules on insurance eligibility (including age, sex, residence, and income) and medical treatment details (diseases and procedures) [16]. The NHIS official review committee approved this study (Protocol Number: NHIS‐2022‐1‐240) and permitted access to the data.

### 2.2. Study Population and Design

We included adult patients (>18 years) who received CRRT for at least 3 days between January 1, 2010, and December 31, 2019 (*n* = 119,421). We excluded patients with preexisting end‐stage kidney disease (*n* = 19,605) or preexisting diabetes (*n* = 53,728) and those discharged or died within 7 days of admission (*n* = 66,415) as the definition of hyperglycemia relied on the use of oral hypoglycemic agents (OHAs) or insulin beyond 7 days; these criteria were not mutually exclusive, as individual patients could meet more than one exclusion criterion. After applying all exclusion criteria, 26,935 patients remained. Of these, 11,013 surviving patients discharged from hospitals were included in the final analysis.

### 2.3. Ethics Approval and Consent to Participate

The study was approved by the Institutional Review Board of Samsung Medical Center (IRB Number 2021‐01‐052) in accordance with the Declaration of Helsinki of 1975. The Institutional Review Board of Samsung Medical Center waived the requirement for informed consent due to the retrospective nature of the study and de‐identified data collection.

### 2.4. Exposure

Hyperglycemia was defined as receipt of OHA or insulin treatment for at least 7 days during hospitalization, identified using medication codes, serving as a clinical marker of recognized hyperglycemia requiring intervention during critical illness. As patients with preexisting diabetes were excluded from the study, this treatment‐based approach represents an operational definition of stress hyperglycemia in a nondiabetic population. The rationale for this treatment‐based definition was that the NHIS database, which is primarily designed for claims and reimbursement purposes, does not contain detailed clinical laboratory data, including blood glucose values or HbA1c levels.

### 2.5. Study Variables

The NHIS utilizes coding based on the 10th revision of the International Statistical Classification of Diseases (ICD‐10) for inpatient and outpatient encounters, procedures, and prescriptions [17]. These data are considered reliable due to routine audits and frequent use in research publications [16, 18]. We identified comorbidities using ICD‐10 codes, including preexisting chronic liver disease, chronic kidney disease, and cancer within the past year prior to hospitalization. The Charlson index additionally summarized these comorbidities [19, 20]. We also included hypertension (ICD‐10 code: I10–I13 and I15) and septic shock (≥2 days of vasopressor and ≥1 week of antibiotics). Management procedures included CRRT (Korean NHI procedure codes O7031–O7035 and O7051–O7055), mechanical ventilation (Korean NHI procedure codes M5857, M5858, or M5860), extracorporeal membrane oxygenation (ECMO)/intra aorta balloon pump (IABP) (Korean NHI procedure codes O1901–O1904, O1921, and O1922), and high‐dose steroids (i.e., ≥7 consecutive administrations of intravenous methylprednisolone ≥40 mg, or intravenous dexamethasone ≥40 mg; ≥35 mg of oral prednisolone, ≥28 mg of oral methylprednisolone, or ≥42 mg deflazacort; or ≥56 mg of oral dexamethasone).

### 2.6. Outcomes

Primary outcomes were all‐cause mortality and adverse cardiovascular events (CVEs) after discharge. The secondary outcome was new‐onset diabetes diagnosed after 1 year. Vital status was obtained through death certification data from Statistics of South Korea [16]. CVE was defined as a hospitalization with a diagnosis of stroke (ICD‐10 codes I63, I64, and G45), heart failure (ICD‐10 codes I110, I130, I132, I255, I420, I425–I429, I43, I50, and I971), acute myocardial infarction (MI) (ICD‐10 codes I21–I23 and I252), or any revascularization procedure code (percutaneous coronary intervention [PCI], Korean National Health Insurance codes M6551–M6554, M6561–M6567, and M6571–M6572; or coronary artery bypass graft surgery [CABG], O1640–O1642, O1647–O1649, OA640–OA642, and OA647–OA649). New‐onset diabetes, diagnosed at least 1 year after discharge, was defined by at least two occurrences of ICD‐10 codes E11–E14 along with a diabetes medication code (including biguanides, sulfonylurea, meglitinides, thiazolidinediones, dipeptidyl peptidase‐4 inhibitors, α‐glucosidase inhibitors, sodium–glucose cotransporter 2 inhibitors, insulin, or glucagon‐like peptide 1 agonist).

### 2.7. Statistical Analysis

Continuous variables were presented as mean and standard deviation or median and interquartile range. These were compared using the *t*‐test or Mann–Whitney tests. Categorical variables were presented as frequencies and percentages and compared using a chi‐square test or Fisher’s exact test.

To identify factors associated with receiving OHA or insulin during hospitalization (among nondiabetic patients), we performed a multivariable logistic regression analysis. We selected relevant variables based on a literature review by an expert and ensured they were not statistically interrelated.

We used survival analysis to assess the impact of hyperglycemia treatment on mortality and major CVEs after discharge. We followed up with patients who survived hospitalization, calculating person‐time from discharge to the event (CVE, all‐cause mortality, or end of follow‐up). Kaplan–Meier curves were used to estimate survival probabilities, compared by log‐rank tests. Cox proportional hazards models estimated hazard ratios (HR) with 95% confidence intervals (CIs) for both outcomes, considering age, sex, type of hospital (tertiary vs. non‐tertiary), Charlson index, cardiovascular diseases, chronic pulmonary disease, renal disease, moderate or severe liver disease, malignancy, hypertension, CRRT duration, revascularization, ECMO/IABP/radiofrequency catheter ablation (RFCA), mechanical ventilation, and high‐dose steroid. Considering the correlation of medical condition and its treatment, we performed multivariable analysis by excluding PCI/CABG/RFCA, mechanical ventilation, and ECMO/IABP in Model 1 and excluding cardiogenic and septic shock in Model 2. The proportional hazards assumption was assessed using Schoenfeld residual tests, with both global and exposure‐specific test statistics calculated for each multivariable Cox model. No significant violations were detected in either the all‐cause mortality model (global test: *p* = 0.61; exposure‐specific test for stress hyperglycemia: *p* = 0.72) or the composite cardiovascular outcome model (global test: *p* = 0.49; exposure‐specific test: *p* = 0.65). Finally, we analyzed the incidence of new‐onset diabetes only in patients surviving at least 1 year after discharge and not receiving diabetes treatment within 1 year. Person‐time was calculated from 1 year after discharge to diabetes diagnosis, all‐cause mortality, or end of follow‐up. All analyses were two‐sided, with *p*‐values < 0.05 considered statistically significant. We used SAS Version 9.2 (SAS Institute, Inc., Cary, NC, USA) and R software Version 3.3.2 (Free Software Foundation, Inc., Boston, MA, USA) for statistical analyses.

## 3. Results

### 3.1. Characteristics of Surviving Patients

Of the 11,013 survivors discharged, 8604 (78.1%) were classified as the control group, while 2409 (21.9%) were in the hyperglycemia group. Table 1 summarizes the characteristics of these discharged patients. The hyperglycemia group was younger but had a higher prevalence of hypertension and shock. Conversely, they had a lower prevalence of comorbidities, including cardiovascular disease, chronic pulmonary disease, renal disease, liver disease, and malignancy compared to the control group. Additionally, the hyperglycemia group received more PCI/CABG/RFCA, mechanical ventilation, ECMO/IABP, and high‐dose steroids during hospitalization.

**Table 1:** Characteristics of patients who survived and were discharged.

Characteristics	Overall (*N* = 11013)	Control (*N* = 8604)	Hyperglycemia (*N* = 2409)	*p*‐Value
Age (years)	65.2 ± 16.8	67.2 ± 16.4	66.5 ± 14.7	<0.001
Sex (male)	4305 (39.1)	3365 (39.1)	940 (39.0)	0.94
Tertiary	5835 (53.0)	4591 (53.4)	1244 (51.6)	0.14
Charlson index	1.7 (1.7)	2.0 (2.0)	2.0 (1.8)	<0.001
Cardiovascular disease	4546 (41.3)	3616 (42.0)	930 (38.6)	0.003
Chronic pulmonary disease	3568 (32.4)	2883 (33.5)	685 (28.4)	<0.001
Renal disease	1477 (13.4)	1229 (14.3)	248 (10.3)	<0.001
Moderate or severe liver disease	397 (3.6)	338 (3.9)	59 (2.5)	<0.001
Malignancy	1312 (11.9)	1138 (13.2)	174 (7.2)	<0.001
Hypertension	6546 (59.4)	4937 (57.4)	1609 (66.8)	<0.001
Cardiogenic shock	508 (4.6)	305 (3.5)	203 (8.4)	<0.001
Septic shock	6276 (57.0)	4690 (54.5)	1586 (65.8)	<0.001
Treatment				
CRRT duration (days)	6.3 ± 4.6	7.3 ± 5.8	8.0 ± 6.9	0.11
PCI/CABG/RFCA	564 (5.1)	338 (3.9)	226 (9.4)	<0.001
Mechanical ventilation	5044 (45.8)	3699 (43.0)	1345 (55.8)	<0.001
ECMO/IABP	381 (3.5)	269 (3.1)	112 (4.7)	<0.001
High‐dose steroid	1340 (12.2)	1021 (11.9)	319 (13.2)	0.07

*Note:* Values were presented as number (%) or mean (standard deviation).

Abbreviations: CABG, coronary artery bypass graft; CRRT, continuous renal replacement therapy; ECMO, extracorporeal membrane oxygenation; IABP, intra aorta balloon pump; PCI, percutaneous coronary intervention; RFCA, radiofrequency catheter ablation.

### 3.2. Mortality

In‐hospital mortality was lower in the hyperglycemia group than in the control group (53.4% vs. 60.5%, *p* < 0.001). After discharge, the overall mortality rates were also slightly lower in the hyperglycemia group (13.7 per 100 person‐years) than in the control group (15.6 per 100 person‐years), as shown in Figure 1A. Furthermore, surviving patients with hyperglycemia during hospitalization had a statistically significant decrease in risk of all‐cause mortality after discharge (adjusted HR 0.92, 95% CI 0.86–0.99, Table 2).

Figure 1Cumulative incidence of all‐cause death (A) and cardiac composite outcomes (B) in patients who survived and were discharged.

**Figure:**
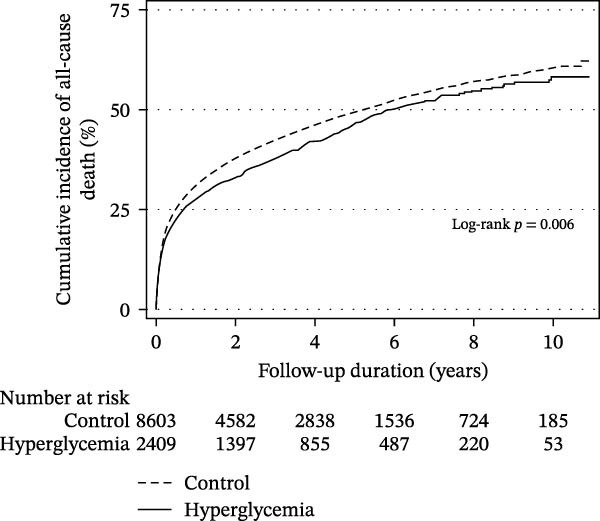
(A)

**Figure:**
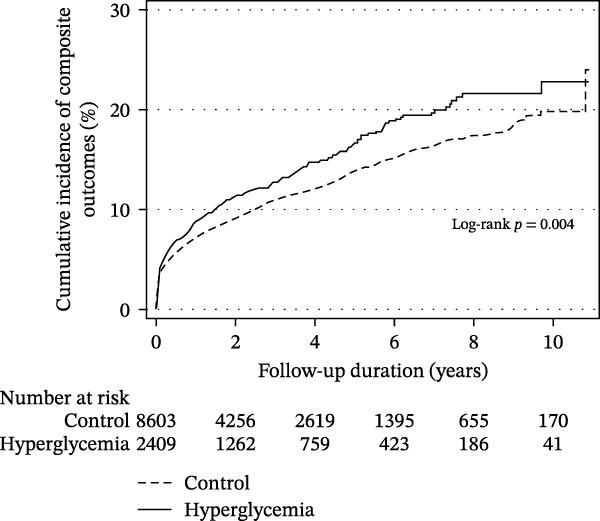
(B)

**Table 2:** Stress hyperglycemia and clinical outcomes after discharge.

Outcomes	Control (*n* = 8604)	Hyperglycemia^a^ (*n* = 2409)	Crude	Adjusted^b^	
	No. (per 1000 person‐years)		HR (95% CI)	HR (95% CI)	*p*
All‐cause mortality	4156 (15.6)	1092 (13.7)	0.90 (0.83–0.97)	0.92 (0.86–0.99)	0.020
Cardiovascular events	796 (3.3)	294 (4.1)	1.22 (1.07–1.40)	1.00 (0.87–1.15)	1.00
Ischemic stroke	388 (1.5)	138 (1.8)	1.22 (1.04–1.44)	1.07 (0.91–1.27)	0.42^d^
Heart failure	338 (1.3)	73 (0.9)	0.89 (0.74–1.08)	0.94 (0.73–1.20)	0.62^d^
Acute myocardial infarction	93 (0.4)	75 (1.0)	2.47 (1.86–3.28)	1.53 (1.11–2.12)	0.010^d^
Revascularization	135 (0.5)	78 (1.0)	1.97 (1.45–2.67)	1.41 (1.02–1.94)	0.036^d^
New‐onset diabetes (*n* = 5100)^c^	39 (0.2)	109 (1.9)	9.29 (6.42–13.44)	10.10 (6.92–14.73)	<0.001

Abbreviations: CI, confidence interval; HR, hazard ratio.

^a^Hyperglycemia was defined as oral hypoglycemic agents or insulin treatment for at least 7 days during hospitalization.

^b^Adjusted for age, sex, tertiary, Charlson index, hypertension, CRRT duration, revascularization, mechanical ventilation, ECMO/IABP, and high‐dose steroid.

^c^New‐onset diabetes was analyzed among patients who survived 1 year after discharge, excluding cases that occurred within 1 year of discharge (*N* = 5100).

^d^Bonferroni correction was applied across the four individual cardiovascular outcomes, yielding a corrected significance threshold of *p* < 0.0125.

### 3.3. Cardiovascular Outcomes

After discharge, the incidence of CVE was slightly higher in the hyperglycemia group (4.1 per 100 person‐years) than in the control group (3.3 per 100 person‐years), as shown in Figure 1B. Although initial analysis suggested an association between hyperglycemia and increased CVE risk, this association became nonsignificant after adjusting for confounding factors (multivariable analysis: adjusted HR 1.00, 95% CI 0.87–1.15, Table 2). However, within CVEs, after applying Bonferroni correction across the four individual cardiovascular outcomes (corrected threshold *p* < 0.0125), hyperglycemia remained linked to a higher risk of new‐onset acute MI (adjusted HR 1.53, 95% CI 1.11–2.12, *p* = 0.010). Although the risk of coronary revascularization procedures was also elevated (adjusted HR 1.41, 95% CI 1.02–1.94, *p* = 0.036), this did not reach the Bonferroni‐corrected significance threshold. (Table 2). When we applied false discovery rate control using the Benjamini–Hochberg procedure, the association with acute MI remained the most robust signal with an adjusted value around 0.04, whereas the association with revascularization was attenuated and did not clearly meet the adjusted threshold.

### 3.4. New‐Onset Diabetes

**Figure 2:**
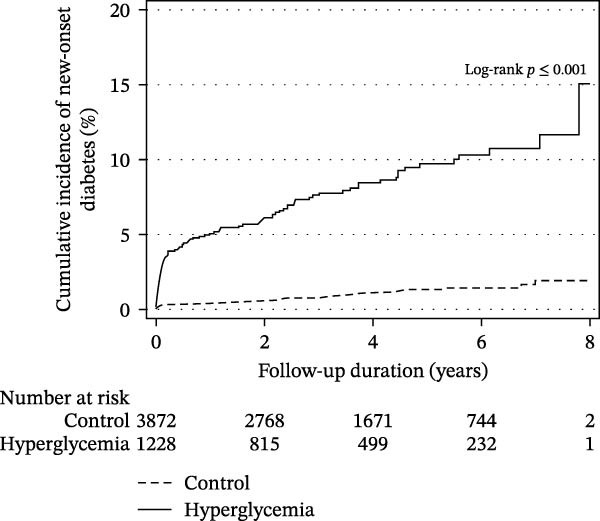
Cumulative incidence of new‐onset diabetes in patients who survived 1 year after discharge.

Among patients surviving at least 1 year after discharge, excluding those diagnosed with diabetes within the first year (*N* = 5100), the hyperglycemia group had a consistently higher rate of new‐onset diabetes throughout the follow‐up period. The incidence rates were 0.2 and 1.9 per 100 person‐years in the control and hyperglycemia groups, respectively (Figure 2). Hyperglycemia during hospitalization remained significantly associated with a 10.10‐fold increased risk of developing diabetes after 1 year (adjusted HR 10.10, 95% CI 6.92–14.73) (Table 2).

### 3.5. Factors Associated With Stress Hyperglycemia During Hospitalization Among All Patients Including In‐Hospital Deaths

Among 26,935 patients with AKI receiving CRRT who did not have preexisting diabetes, 5166 (19.2%) developed hyperglycemia during hospitalization. Details on patient characteristics according to hyperglycemia status are provided in Table S1. After adjusting for covariates, the following conditions were found to be associated with an increased risk of hyperglycemia during hospitalization: age < 65 years (adjusted odds ratio [aOR] 1.13, 95% CI 1.05–1.21), hypertension (aOR 1.49, 95% CI 1.38–1.60), cardiogenic shock (aOR 1.79, 95% CI 1.58–2.04), and septic shock (aOR 1.48, 95% CI 1.37–1.61). In contrast, except for hypertension, other comorbid conditions were associated with a decreased risk of hyperglycemia. The analysis also revealed associations between hyperglycemia and the following treatment factors: longer CRRT duration (aOR 1.01, 95% CI 1.00–1.01), procedures such as PCI/CABG/RFCA (aOR 1.82, 95% CI 1.59–2.09), mechanical ventilation (aOR 1.42, 95% CI 1.31–1.53), and high‐dose steroid use (aOR 2.09, 95% CI 1.93–2.26) (Table 3).

**Table 3:** Risk factors for hyperglycemia during hospitalization among all patients including in‐hospital deaths.

Characteristics	Crude	Model 1	Model 2
	OR (95% CI)	OR (95% CI)	OR (95% CI)
Age less than 65 years	1.04 (0.98–1.11)	1.13 (1.05–1.21)	1.11 (1.03–1.19)
Sex (male)	0.96 (0.90–1.02)	0.99 (0.93–1.06)	0.97 (0.91–1.04)
Tertiary	1.14 (0.95–1.36)	1.15 (0.96–1.87)	1.13 (0.95–1.36)
Comorbidity			
Charlson index	1.00 (0.98–1.02)	1.14 (1.11–1.16)	1.13 (1.11–1.16)
Cardiovascular disease	0.88 (0.82–0.94)	0.78 (0.72–0.83)	0.78 (0.73–0.84)
Chronic pulmonary disease	0.85 (0.80–0.91)	0.80 (0.75–0.86)	0.80 (0.75–0.86)
Renal disease	0.74 (0.67–0.82)	0.65 (0.58–0.72)	0.65 (0.58–0.72)
Moderate or severe liver disease	0.51 (0.44–0.59)	0.35 (0.29–0.41)	0.36 (0.31–0.43)
Malignancy	0.67 (0.61–0.73)	0.41 (0.36–0.46)	0.43 (0.38–0.48)
Hypertension	1.26 (1.18–1.34)	1.49 (1.38–1.60)	1.50 (1.39–1.62)
Cardiogenic shock	2.15 (1.90–2.42)	1.79 (1.58–2.04)	—
Septic shock	1.51 (1.39–1.63)	1.48 (1.37–1.61)	—
Treatment			
CRRT duration (days)	1.02 (1.01–1.02)	1.01 (1.00–1.01)	1.01 (1.00–1.01)
PCI/CABG/RFCA	2.18 (1.93–2.45)	—	1.82 (1.59–2.09)
Mechanical ventilation	1.54 (1.43–1.65)	—	1.42 (1.31–1.53)
ECMO/IABP	1.65 (1.46–1.85)	—	1.03 (0.90–1.18)
High‐dose steroid	1.91 (1.77–2.06)	2.12 (1.96–2.30)	2.09 (1.93–2.26)

Abbreviations: CABG, coronary artery bypass graft; CI, confidence interval; CRRT, continuous renal replacement therapy; ECMO, extracorporeal membrane oxygenation; IABP, intra aorta balloon pump; PCI, percutaneous coronary intervention; RFCA, radiofrequency catheter ablation.

## 4. Discussion

In this nationwide cohort study of critically ill patients with AKI requiring CRRT, we found that stress hyperglycemia, defined as OHA or insulin treatment during hospitalization, was associated with a decreased risk of both short‐term and long‐term mortality. However, it was also associated with increased risks of acute MI, coronary revascularization procedures, and new‐onset diabetes after discharge. Hypertension, cardiogenic or septic shock, longer CRRT duration, PCI/CABG/RFCA, mechanical ventilation, and high‐dose steroid use were associated with an increased risk of stress hyperglycemia, while other underlying medical conditions were linked to a lower risk.

The association of hyperglycemia with better survival in our study is counterintuitive to the established view of hyperglycemia as detrimental [2, 3]. Stress hyperglycemia might reflect an appropriate response to critical illness, potentially indicating the attempt of the body to ensure glucose utilization despite insulin resistance [3]. Overly aggressive glucose control can lead to increased mortality due to hypoglycemia [21, 22]. While earlier studies showed the benefits of strict glycemic control in surgical intensive care unit patients [23], a recent study suggests that mild hyperglycemia might be linked to lower mortality in sepsis [24]. As our analysis could not incorporate the degree of hyperglycemia, further research is needed to clarify this association and determine the overall prognostic impact of hyperglycemia in patients requiring CRRT.

Our study revealed that underlying comorbidities, except hypertension, were linked to a lower risk of stress hyperglycemia. While the exact reason behind this inverse relationship remains unclear, stress hyperglycemia might represent a beneficial adaptation to acute illness. This compensatory response could potentially be inadequate in patients with preexisting medical conditions [25]. Moreover, hyperglycemia during hospitalization was associated with a higher risk of future MI or coronary revascularization procedures, but not with ischemic stroke or heart failure. The established link between insulin resistance and cardiovascular disease is well‐documented [26], with connections to both atherosclerosis [27] and endothelial dysfunction [28]. Therefore, the association with coronary artery disease is unsurprising. However, the link between insulin resistance and ischemic stroke or heart failure is also known [29, 30]. It is unclear why hyperglycemia during hospitalization was not linked to these specific complications in our study. This finding suggests that critically ill patients undergoing CRRT with hyperglycemia might benefit from targeted screening for ischemic heart disease after discharge.

Critically ill patients undergoing CRRT with hyperglycemia during hospitalization exhibited a significantly increased risk of new‐onset diabetes. To minimize confounding factors like acute illness and selection bias from early mortality, we only included patients surviving at least 1 year after discharge. Our findings align with previous research demonstrating a connection between hyperglycemia during critical illness and the risk of future diabetes [1, 31]. Similar associations have been observed in patients with AKI requiring renal replacement therapy [14]. This association may be explained by several possible mechanisms. Patients with undiagnosed diabetes or prediabetes may already be enriched in the hyperglycemia subgroup, thereby contributing to the observed high incidence of new‐onset diabetes. Furthermore, high‐dose steroid therapy may have substantially contributed to the development of diabetes. Systemic administration of steroids is well known as a cause of hyperglycemia and diabetes [32]. One study reported that up to 70% of nondiabetic patients developed in‐hospital hyperglycemia when treated with high‐dose steroids [33]. However, evidence that steroid therapy during acute illness increases the long‐term risk of diabetes remains limited, and further investigation is warranted. Additionally, surveillance bias may have contributed to the observed higher incidence of new‐onset diabetes in the hyperglycemia group, as patients who received glucose‐lowering treatment during hospitalization may have been more closely monitored for metabolic complications after discharge. Nevertheless, the presence of stress hyperglycemia requiring pharmacologic treatment during critical illness retains significant clinical importance as an indicator for identifying high‐risk patients. Our finding highlighted the importance of active screening for and managment 8 of new‐onset diabetes in patients requiring CRRT who experience hyperglycemia during hospitalization.

Our study has several limitations. First, the retrospective nature of the study limited our ability to definitively establish causality between hyperglycemia and outcomes. Additionally, the Korean NHIS database lacks detailed information on patient conditions or laboratory results, potentially leaving out confounding factors. Furthermore, although we adjusted for the Charlson comorbidity index, which includes cardiovascular disease, we did not separately adjust for preexisting coronary artery disease or prior revascularization history. Given that baseline coronary artery disease is a strong predictor of future revascularization procedures, this may have confounded the observed association between stress hyperglycemia and coronary revascularization after discharge. Second, our definition of stress hyperglycemia relied on OHA/insulin prescriptions due to unavailable blood glucose data in the NHIS database. This treatment‐based definition may be susceptible to information and misclassification bias, as the decision to prescribe glucose‐lowering medications can vary based on hospital policies, physician preferences for glycemic targets, and individual clinical judgment. Consequently, this approach may underestimate or overestimate the true prevalence of hyperglycemia and affect the magnitude of associations observed. Additionally, this definition inevitably resulted in the grouping of patients with varying degrees of hyperglycemia into a single category. Third, to minimize population heterogeneity, we exclusively focused on critically ill patients with AKI requiring CRRT. Therefore, our findings might not apply to all critically ill patients with AKI. Despite these limitations, our study offers valuable clinical insights by demonstrating a clear association between significant stress hyperglycemia and long‐term outcomes in a large cohort of critically ill patients requiring CRRT for severe AKI.

## 5. Conclusion

In critically ill patients with AKI undergoing CRRT, stress hyperglycemia during hospitalization was associated with increased risks of future coronary events and new‐onset diabetes. This underscores the importance of implementing proactive screening and close follow‐up for ischemic heart disease and diabetes in these high‐risk patients who survive hospitalization and experience stress hyperglycemia.

## Author Contributions

Hye Ryoun Jang conceptualized the study and provided supervision. Hyejeong Park and Danbee Kang were responsible for data curation. Junseok Jeon, Minhyung Kim, Kyungho Lee, Danbee Kang, Juhee Cho, Jung Eun Lee, Wooseong Huh, and Hye Ryoun Jang were responsible for investigation. Hyejeong Park and Danbee Kang were responsible for formal analysis. Junseok Jeon, Hyejeong Park, Danbee Kang, Juhee Cho, and Hye Ryoun Jang were responsible for methodology. Hyejeong Park and Danbee Kang were responsible for visualization. Junseok Jeon and Danbee Kang wrote the original draft. Junseok Jeon and Hye Ryoun Jang reviewed and edited the manuscript. Juhee Cho, Jung Eun Lee, and Wooseong Huh supervised the study. All authors reviewed the manuscript.

## Funding

This research was supported by a grant of Patient‐Centered Clinical Research Coordinating Center (PACEN) funded by the Ministry of Health and Welfare, Republic of Korea (Grant RS‐2024‐00340973).

## Disclosure

An earlier version of this study was previously presented as a poster in the “Clinical, Outcomes, and Trials – Epidemiology and Pathophysiology” session at the 2024 American Society of Nephrology (ASN) Kidney Week, held in San Diego, California, USA, from November 5 to 10, under the title “Stress Hyperglycemia and Long‐term Outcomes in Patients with AKI Requiring Continuous Kidney Replacement Therapy.” The funder had no role in the study design, data collection, analysis, reporting, or the decision to submit the manuscript for publication.

## Conflicts of Interest

The authors declare no conflicts of interest.

## Supporting Information

Additional supporting information can be found online in the Supporting Information section.

## Supporting information


**Supporting Information** Table S1: Characteristics of patients with acute kidney injury who received continuous renal replacement therapy who did not have preexisting diabetes.

## Data Availability

The data that support the findings of this study are available from the Korean National Health Insurance Service. Restrictions apply to the availability of these data, which were used under license for this study. Data are available from https://nhiss.nhis.or.kr/bd/ab/bdaba000eng.do with the permission of the Korean National Health Insurance Service.

## References

[1] Ali Abdelhamid Y. , Kar P. , and Finnis M. E. , et al.Stress Hyperglycaemia in Critically Ill Patients and the Subsequent Risk of Diabetes: A Systematic Review and Meta-Analysis, Critical Care. (2016) 20, no. 1, 10.1186/s13054-016-1471-6, 2-s2.0-84992107988, 301. 27677709 PMC5039881

[2] Umpierrez G. E. , Isaacs S. D. , Bazargan N. , You X. , Thaler L. M. , and Kitabchi A. E. , Hyperglycemia: An Independent Marker of In-Hospital Mortality in Patients With Undiagnosed Diabetes, The Journal of Clinical Endocrinology and Metabolism. (2002) 87, no. 3, 978–982, 10.1210/jcem.87.3.8341, 2-s2.0-0036962051. 11889147

[3] Krinsley J. S. , Association Between Hyperglycemia and Increased Hospital Mortality in a Heterogeneous Population of Critically Ill Patients, Mayo Clinic Proceedings. (2003) 78, no. 12, 1471–1478, 10.4065/78.12.1471, 2-s2.0-0345374590. 14661676

[4] Capes S. E. , Hunt D. , Malmberg K. , and Gerstein H. C. , Stress Hyperglycaemia and Increased Risk of Death After Myocardial Infarction in Patients With and Without Diabetes: A Systematic Overview, The Lancet. (2000) 355, no. 9206, 773–778, 10.1016/S0140-6736(99)08415-9, 2-s2.0-0034603545. 10711923

[5] Inzucchi S. E. , Management of Hyperglycemia in the Hospital Setting, New England Journal of Medicine. (2006) 355, no. 18, 1903–1911, 10.1056/NEJMcp060094, 2-s2.0-33750500326. 17079764

[6] Mehta R. L. , Glycemic Control and Critical Illness, Journal of the American Society of Nephrology. (2007) 18, no. 10, 2623–2627, 10.1681/ASN.2007010109, 2-s2.0-34948882675. 17656475

[7] Meyer C. , Dostou J. M. , and Gerich J. E. , Role of the Human Kidney in Glucose Counterregulation, Diabetes. (1999) 48, no. 5, 943–948, 10.2337/diabetes.48.5.943, 2-s2.0-0032915448. 10331396

[8] Carreira A. , Castro P. , Mira F. , Melo M. , Ribeiro P. , and Santos L. , Acute Kidney Injury: A Strong Risk Factor for Hypoglycaemia in Hospitalized Patients With Type 2 Diabetes, Acta Diabetologica. (2023) 60, no. 9, 1179–1185, 10.1007/s00592-023-02112-0. 37173530 PMC10359379

[9] Vriesendorp T. M. , van Santen S. , and DeVries J. H. , et al.Predisposing Factors for Hypoglycemia in the Intensive Care Unit, Critical Care Medicine. (2006) 34, no. 1, 96–101, 10.1097/01.CCM.0000194536.89694.06, 2-s2.0-29744458366. 16374162

[10] Basi S. , Pupim L. B. , and Simmons E. M. , et al.Insulin Resistance in Critically Ill Patients With Acute Renal Failure, American Journal of Physiology-Renal Physiology. (2005) 289, no. 2, F259–F264, 10.1152/ajprenal.00002.2005, 2-s2.0-22344441558. 15840772

[11] Li M. , Deng C. M. , and Su X. , et al.Hyperglycemia is Associated With Worse 3-Year Survival in Older Patients Admitted to the Intensive Care Unit After Non-Cardiac Surgery: Post Hoc Analysis of a Randomized Trial, Frontiers in Medicine. (2022) 9, 10.3389/fmed.2022.1003186, 1003186. 36579147 PMC9790906

[12] Upur H. , Li J. L. , and Zou X. G. , et al.Short and Long-Term Prognosis of Admission Hyperglycemia in Patients With and Without Diabetes After Acute Myocardial Infarction: A Retrospective Cohort Study, Cardiovascular Diabetology. (2022) 21, no. 1, 10.1186/s12933-022-01550-4, 114. 35739511 PMC9229884

[13] Plummer M. P. , Finnis M. E. , and Phillips L. K. , et al.Stress Induced Hyperglycemia and the Subsequent Risk of Type 2 Diabetes in Survivors of Critical Illness, PLoS ONE. (2016) 11, no. 11, 10.1371/journal.pone.0165923, 2-s2.0-84994424524. PMC5100960 27824898

[14] Lin Y. F. , Lin S. L. , and Huang T. M. , et al.New-Onset Diabetes After Acute Kidney Injury Requiring Dialysis, Diabetes Care. (2018) 41, no. 10, 2105–2110, 10.2337/dc17-2409, 2-s2.0-85056523998. 30104297

[15] Kim D. , Yang P. S. , and You S. C. , et al.Treatment Timing and the Effects of Rhythm Control Strategy in Patients With Atrial Fibrillation: Nationwide Cohort Study, BMJ. (2021) 373, 10.1136/bmj.n991, n991. 33975876 PMC8111568

[16] Lee J. , Lee J. S. , Park S.-H. , Shin S. A. , and Kim K. W. , Cohort Profile: The National Health Insurance Service-National Sample Cohort (NHIS-NSC), South Korea, International Journal of Epidemiology. (2017) 46, no. 2, 10.1093/ije/dyv319, 2-s2.0-85053001577, e15. 26822938

[17] Chun C. B. , Kim S. Y. , Lee J. Y. , Lee S. Y. , and World Health Organization , Republic of Korea: Health System Review, 2009.

[18] Shin D. W. , Cho B. L. , and Guallar E. , Korean National Health Insurance Database, JAMA Internal Medicine. (2016) 176, no. 1, 10.1001/jamainternmed.2015.7110, 2-s2.0-85047290381, 138. 26747667

[19] Charlson M. E. , Pompei P. , Ales K. L. , and MacKenzie C. R. , A New Method of Classifying Prognostic Comorbidity in Longitudinal Studies: Development and Validation, Journal of Chronic Diseases. (1987) 40, no. 5, 373–383, 10.1016/0021-9681(87)90171-8, 2-s2.0-0023092594. 3558716

[20] Kim K. H. , Comparative Study on Three Algorithms of the ICD-10 Charlson Comorbidity Index With Myocardial Infarction Patients, Journal of Preventive Medicine and Public Health. (2010) 43, no. 1, 42–49, 10.3961/jpmph.2010.43.1.42, 2-s2.0-77953664802. 20185982

[21] Finfer S. , Chittock D. R. , and Su S. Y. , et al.Intensive Versus Conventional Glucose Control in Critically Ill Patients, The New England Journal of Medicine. (2009) 360, no. 13, 1283–1297, 10.1056/NEJMoa0810625, 2-s2.0-63249128249. 19318384

[22] Brunkhorst F. M. , Engel C. , and Bloos F. , et al.Intensive Insulin Therapy and Pentastarch Resuscitation in Severe Sepsis, New England Journal of Medicine. (2008) 358, no. 2, 125–139, 10.1056/NEJMoa070716, 2-s2.0-38049096093. 18184958

[23] van den Berghe G. , Wouters P. , and Weekers F. , et al.Intensive Insulin Therapy in Critically Ill Patients, New England Journal of Medicine. (2001) 345, no. 19, 1359–1367, 10.1056/NEJMoa011300, 2-s2.0-0035829852. 11794168

[24] Tiruvoipati R. , Chiezey B. , and Lewis D. , et al.Stress Hyperglycemia May not be Harmful in Critically Ill Patients With Sepsis, Journal of Critical Care. (2012) 27, no. 2, 153–158, 10.1016/j.jcrc.2011.06.011, 2-s2.0-84858622520. 21855283

[25] Marik P. E. and Bellomo R. , Stress Hyperglycemia: An Essential Survival Response!, Critical Care. (2013) 17, no. 2, 10.1186/cc12514, 2-s2.0-84874718741, 305. 23470218 PMC3672537

[26] Laakso M. and Kuusisto J. , Insulin Resistance and Hyperglycaemia in Cardiovascular Disease Development, Nature Reviews Endocrinology. (2014) 10, no. 5, 293–302, 10.1038/nrendo.2014.29, 2-s2.0-84899490570. 24663222

[27] Bornfeldt K. E. and Tabas I. , Insulin Resistance, Hyperglycemia, and Atherosclerosis, Cell Metabolism. (2011) 14, no. 5, 575–585, 10.1016/j.cmet.2011.07.015, 2-s2.0-80455128548. 22055501 PMC3217209

[28] Janus A. , Szahidewicz-Krupska E. , Mazur G. , and Doroszko A. , Insulin Resistance and Endothelial Dysfunction Constitute a Common Therapeutic Target in Cardiometabolic Disorders, Mediators of Inflammation. (2016) 2016, 10, 10.1155/2016/3634948, 2-s2.0-84978732242, 3634948. 27413253 PMC4931075

[29] Ding P. F. , Zhang H. S. , and Wang J. , et al.Insulin Resistance in Ischemic Stroke: Mechanisms and Therapeutic Approaches, Frontiers in Endocrinology. (2022) 13, 10.3389/fendo.2022.1092431, 1092431. 36589857 PMC9798125

[30] Horwich T. B. and Fonarow G. C. , Glucose, Obesity, Metabolic Syndrome, and Diabetes, Journal of the American College of Cardiology. (2010) 55, no. 4, 283–293, 10.1016/j.jacc.2009.07.029, 2-s2.0-74049143053. 20117431 PMC2834416

[31] Gornik I. , Vujaklija-Brajkovic A. , Renar I. P. , and Gasparovic V. , A Prospective Observational Study of the Relationship of Critical Illness Associated Hyperglycaemia in Medical ICU Patients and Subsequent Development of Type 2 Diabetes, Critical Care. (2010) 14, no. 5, 10.1186/cc9272, 2-s2.0-78651233785, R130. 20615210 PMC2945097

[32] Liu X.-X. , Zhu X.-M. , Miao Q. , Ye H.-Y. , Zhang Z.-Y. , and Li Y.-M. , Hyperglycemia Induced by Glucocorticoids in Nondiabetic Patients: A Meta-Analysis, Annals of Nutrition and Metabolism. (2014) 65, no. 4, 324–332, 10.1159/000365892, 2-s2.0-84911497884. 25402408

[33] Fong A. C. and Cheung N. W. , The High Incidence of Steroid-Induced Hyperglycaemia in Hospital, Diabetes Research and Clinical Practice. (2013) 99, no. 3, 277–280, 10.1016/j.diabres.2012.12.023, 2-s2.0-84876172524. 23298665

