# Supplementary material for: Impact of Stress Hyperglycemia on Long‐Term Outcomes in Patients With Acute Kidney Injury Requiring Continuous Renal Replacement Therapy: A Nationwide Cohort Study
Source: J Diabetes Res. 2026 Mar 26;2026:7769851. doi: 10.1155/jdr/7769851 (PMC13140406; doi:10.1155/jdr/7769851)
Supplement: Supplementary file 1 — Supporting Information Table S1: Characteristics of patients with acute kidney injury who received continuous renal replacement therapy who did not have preexisting diabetes. [file JDR-2026-7769851-s001.pdf]

Supplementary Table 1: Characteristics of patients with acute kidney injury who received continuous renal replacement therapy who did not have preexisting diabetes

	Overall (N=26,935)	Control (N=21,769)	Hyperglycemia (N=5,166)	*p*-value
**Age, years**	67.1 ± 16.1	67.2 ± 16.4)	66.5 ± 14.7	0.002
**Age more than 65 years**	16,494 (61.2)	13,388 (61.5)	3,106 (60.1)	0.07
**Sex, male**	16,802 (62.4)	13,625 (62.6)	3,177 (61.5)	0.15
**Tertiary hospital**	14,879 (55.2)	12,036 (55.3)	2,843 (55.0)	0.74
**Charlson index**	2.0 ± 2.0)	2.0 ± 2.0)	2.0 ± 1.8	0.88
Cardiovascular disease	11,342 (42.1)	9,315 (42.8)	2,027 (39.2)	<0.001
Chronic pulmonary disease	10,124 (37.6)	8,353 (38.4)	1,771 (34.3)	<0.001
Renal disease	3,228 (12.0)	2,737 (12.6)	491 (9.5)	<0.001
Moderate or severe liver disease	1,769 (6.6)	1,564 (7.2)	205 (4.0)	<0.001
Malignancy	5,539 (20.6)	4,732 (21.7)	807 (15.6)	<0.001
Hypertension	15,922 (59.1)	12,654 (58.1)	3,268 (63.3)	<0.001
**Cardiogenic shock**	1,327 (4.9)	892 (4.1)	435 (8.4)	<0.001
**Septic shock**	20,359 (75.6)	16,160 (74.2)	4,199 (81.3)	<0.001
**Treatment**				
CRRT duration, days	7.5 ± 6.0	7.3 ± 5.8	8.0 ± 6.9	<0.001
PCI/CABG/RFCA	1,396 (5.2)	936 (4.3)	460 (8.9)	<0.001
Mechanical ventilation	18,113 (67.2)	14,288 (65.6)	3,825 (74.0)	<0.001
ECMO/IABP	1,595 (5.9)	1,156 (5.3)	439 (8.5)	<0.001
High-dose steroid	5,080 (18.9)	3,721 (17.1)	1,359 (26.3)	<0.001
**In-hospital death**	15,922 (59.1)	13,165 (60.5)	2,757 (53.4)	<0.001

Note: Categorical variables were presented as numbers (%) and continuous variables were presented as mean ± standard deviation

Abbreviations: CABG, coronary artery bypass graft; CRRT, continuous renal replacement therapy; ECMO, extracorporeal membrane

oxygenation; IABP, intra aorta balloon pump; PCI, percutaneous coronary intervention; RFCA, radiofrequency catheter ablation
